# There is no difference in outcome between laparoscopic and open surgery for rectal cancer: a systematic review and meta-analysis on short- and long-term oncologic outcomes

**DOI:** 10.1007/s10151-017-1662-4

**Published:** 2017-08-09

**Authors:** M. Pędziwiatr, P. Małczak, M. Mizera, J. Witowski, G. Torbicz, P. Major, M. Pisarska, M. Wysocki, A. Budzyński

**Affiliations:** 1 2nd Department of General Surgery, Jagiellonian University Medical College, Kopernika 21, 31-501 Kraków, Poland; 2 Centre for Research, Training and Innovation in Surgery (CERTAIN Surgery), Kraków, Poland

**Keywords:** Laparoscopy, Total mesorectal excision, Rectal cancer, Circumferential resection margin, Survival, Local recurrence, Meta-analysis

## Abstract

**Background:**

Until recently there has been little data available about long-term outcomes of laparoscopic rectal cancer surgery. But new randomized controlled trials regarding laparoscopic colorectal surgery have been published. The aim of this study was to compare the short- and long-term oncologic outcomes of laparoscopy and open surgery for rectal cancer through a systematic review of the literature and a meta-analysis of relevant RCTs.

**Methods:**

A systematic review of Medline, Embase and the Cochrane library from January 1966 to October 2016 with a subsequent meta-analysis was performed. Only randomized controlled trials with data on circumferential resection margins were included. The primary outcome was the status of circumferential resection margins. Secondary outcomes included lymph node yield, distal resection margins, disease-free and overall survival rates for 3 and 5 years and local recurrence rates.

**Results:**

Eleven studies were evaluated, involving a total of 2018 patients in the laparoscopic group and 1526 patients in the open group. The presence of involved circumferential margins was reported in all studies. There were no statistically significant differences in the number of positive circumferential margins between the laparoscopic group and open group, RR 1.16, 95% CI 0.89–1.50 and no significant differences in involvement of distal margins (RR 1.13 95% CI 0.35–3.66), completeness of mesorectal excision (RR 1.22, 95% CI 0.82–1.82) or number of harvested lymph nodes (mean difference = −0.01, 95% CI −0.89 to 0.87). Disease-free survival rates at 3 and 5 years were not different (*p* = 0.26 and *p* = 0.71 respectively), and neither were overall survival rates (*p* = 0.19 and *p* = 0.64 respectively), nor local recurrence rates (RR 0.88, 95% CI 0.63–1.23).

**Conclusions:**

Laparoscopic surgery for rectal cancer is associated with similar short-term and long-term oncologic outcomes compared to open surgery. The oncologic quality of extracted specimens seems comparable regardless of the approach used.

**Electronic supplementary material:**

The online version of this article (doi:10.1007/s10151-017-1662-4) contains supplementary material, which is available to authorized users.

## Introduction

There has been a constant increase in the incidence of colorectal cancer. Currently it is the most common gastrointestinal malignancy worldwide [1]. Approximately one-third of all large bowel cancers are located in the rectum [1]. So far, the primary treatment option for rectal adenocarcinoma remains surgery, supported by neoadjuvant and adjuvant therapy [2, 3].

Over the last two decades, a trend towards minimally invasive surgery in the treatment of rectal cancer has been observed [4]. In selected patients, laparoscopic surgery has been reported to achieve better short-term outcomes, which include: lower postoperative morbidity, reduced intraoperative blood loss, less pain, faster recovery and better quality of life [5–8]. Although there is much evidence supporting laparoscopy in terms of perioperative parameters, little is known of the influence of this surgical technique on long-term outcomes. It is generally accepted that, from the oncologic perspective, disease-free survival is considered a primary endpoint in the assessment of treatment quality in rectal cancer. The most important surgical factors related to long-term oncologic results are clear resection margins and completeness of mesorectal excision. So far, several randomized trials comparing laparoscopic and open surgery have been conducted. However, in most of them the oncologic outcomes are not set as primary endpoints (thus creating potential bias related to underpowering) or full resection details are not reported. Moreover, the evidence on survival after open versus laparoscopic surgery within a randomized controlled trial (RCT) environment is sparse, with these results from high-quality RCTs only recently published [9–11].

Our aim was to evaluate the effectiveness of laparoscopy and open surgery for rectal cancer by systematically reviewing the available literature and conducting a meta-analysis of RCTs comparing short-term and long-term oncologic outcomes.

## Materials and methods

### Search strategy

In October 2016, a search was conducted by three teams, with two researchers in each, of Medline, Embase and the Cochrane Library, covering a period from January 1966 to October 2016. The search had no language limitations, so that the review would be as comprehensive as possible. A full search strategy for strategy for OVID platform is available in supplement 1. Reference lists of relevant publications were assessed for additional studies. Furthermore, references from other systematic reviews or meta-analyses on the subject were searched.

A study was included when it comprised adult patients, rectal surgery for malignancy and reported on the circumferential resection margin (CRM) status. Only RCTs were included. Studies were excluded if they were not full-text papers, were not RCTs or did not report data on CRM. Studies that fulfilled all the criteria were eligible for further evaluation.

All teams identified and selected citations from the search independently. In case of doubt about inclusion, an attempt was made to reach a consensus within the team. If no consensus was possible, a decision was made by a third member of the group outside that team. Data from included studies were extracted independently by all teams. The study quality and risk of bias was assessed using the Cochrane Collaboration’s tool for assessing risk of bias.

### Outcome measures

The primary outcome measures of this systematic review were involved CRM status. Secondary outcome measures were distal resection margin, completeness of mesorectal excision, total number of harvested lymph nodes, 3-year disease-free, 5-year disease-free and overall survival rate as well as local recurrence rate. The quality of mesorectal dissection was classified according to Nagtegaal et al. [12]. For the purpose of subsequent meta-analysis, similarly to Nagtegaal’s original paper ‘complete’ and ‘nearly complete’ mesorectal excisions were grouped together as ‘complete’ and were compared with ‘incomplete’ mesorectal excisions.

### Statistical analysis

Analysis was performed using RevMan 5.3 (freeware from the Cochrane Collaboration). Statistical heterogeneity and inconsistency were measured using Cochran’s *Q* and *I* ^2^, respectively. Qualitative outcomes from individual studies were analysed to assess individual and pooled risk ratios (RR) with pertinent 95% confidence intervals (CI) favouring the minimally invasive approach over open surgery and by means of the Mantel–Haenszel fixed-effects method in the presence of low or moderate statistical inconsistency (*I* ^2^ ≤ 10%) and by means of a random-effects method (which better accommodates clinical and statistical variations) in the case of high statistical inconsistency (*I* ^2^ > 10%). For positive outcomes RR was calculated for ‘non-event’ occurrence. When the study included medians and interquartile ranges, we calculated the mean ± standard deviation (SD) using a method proposed by Hozo et al. [13]. Weighted mean differences (WMD) with 95% CI are presented for quantitative variables using the inverse variance fixed-effects or random-effects method. Statistical significance was observed with a two-tailed 0.05 level for hypothesis and with 0.10 for heterogeneity testing, while unadjusted *p* values were reported accordingly. This study was performed according to the Preferred Reporting Items for Systematic Reviews and Meta-Analyses (PRISMA) guidelines.

## Results

**Fig. 1:**
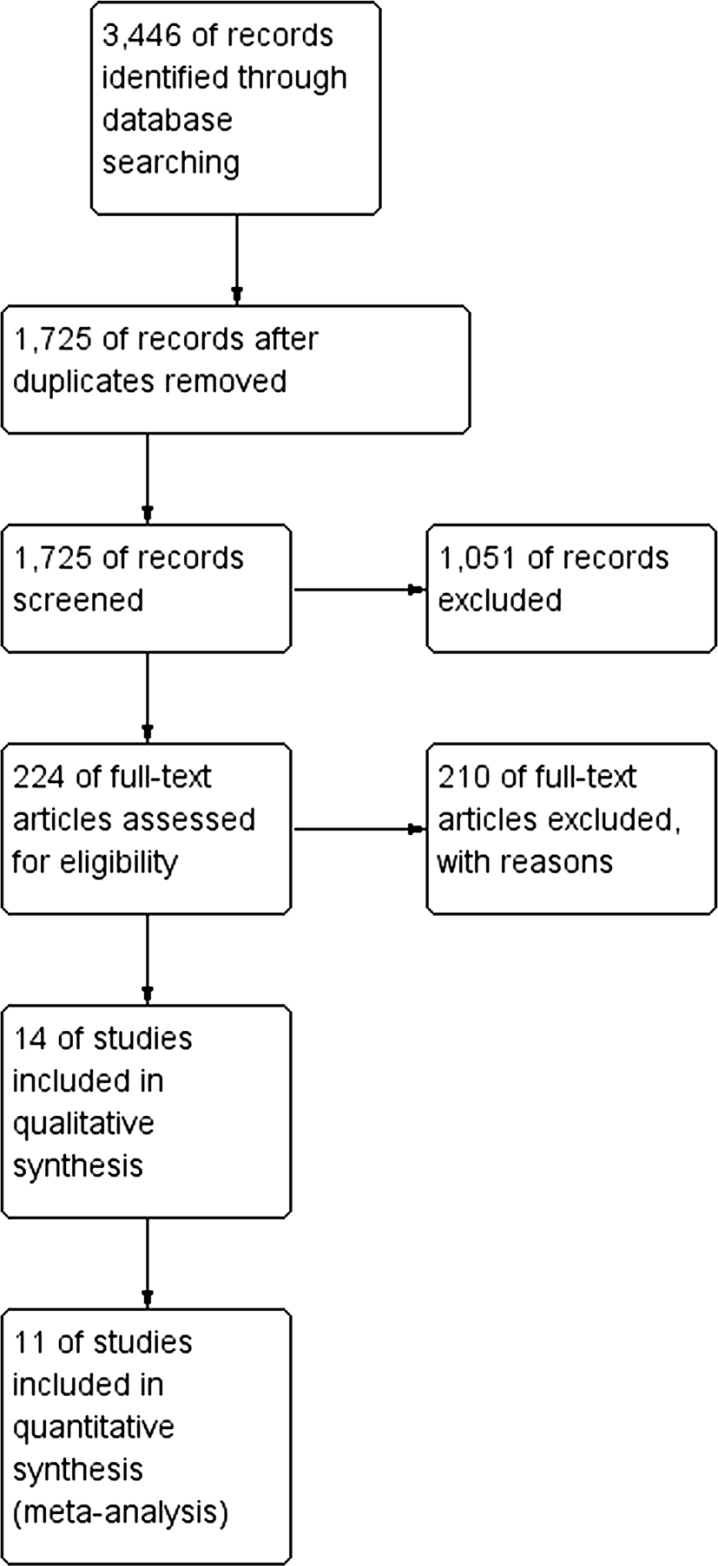
PRISMA flowchart

**Fig. 2:**
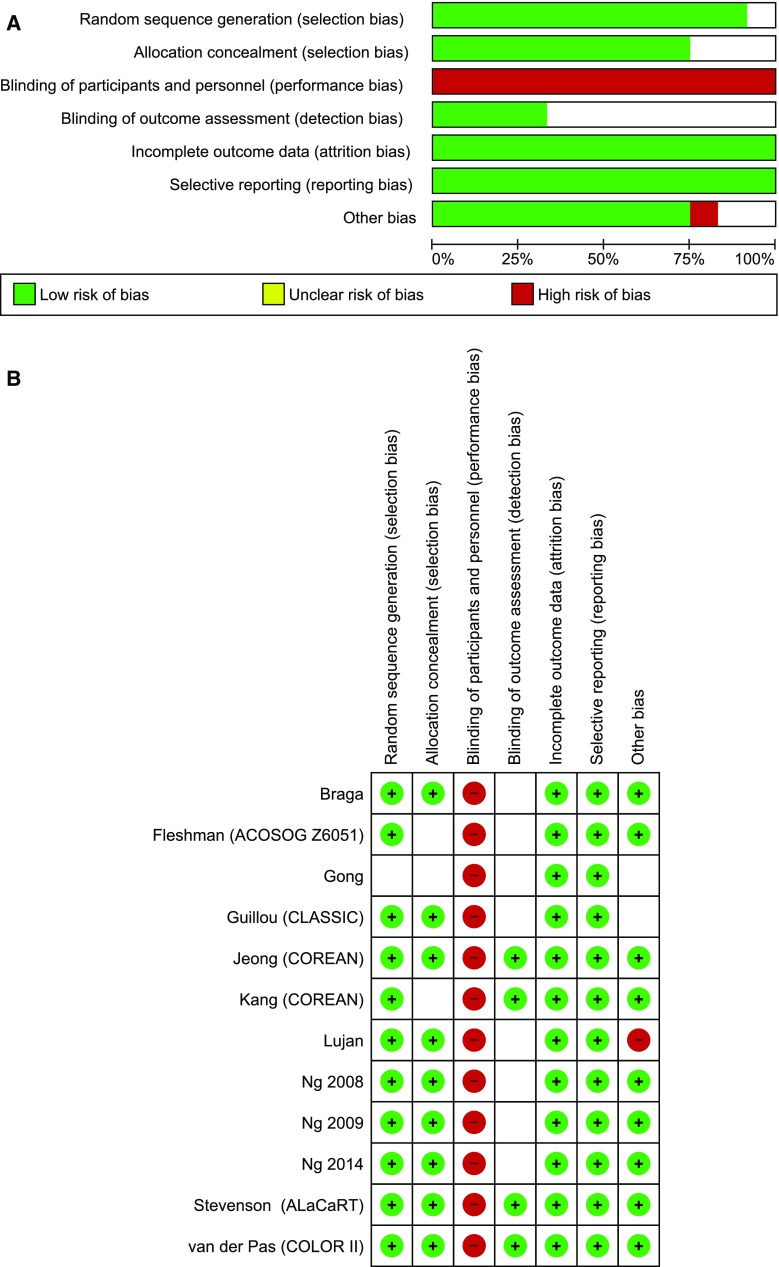
Risk of bias summary

The initial reference search yielded 3446 articles. After removing 1721 duplicates, 1725 articles were evaluated through titles and abstracts. This produced 224 papers suitable for full-text review, of which 14 studies met the eligibility criteria [9–11, 14–24]. There were 3 trials (COLOR II, COREAN, CLASICC) in which results were reported in more than one paper. Papers from the same trial were analysed as one study, so that a total of 11 studies were analysed; 2018 patients in the laparoscopic group and 1526 patients in the open group (Table 1). The literature search and study selection is summarized in Fig. 1. Risk of bias in the studies is assessed in Fig. 2. In general, the risk of bias in the studies was low. Due to the nature of the treatment, the blinding of participants and personnel was impossible to perform. The outcome assessment was the main source of bias as most of the studies did not clearly define how and by whom it was performed. The paper with the most potential for bias, Gong et al. [14], has been included in the analysis as it had little impact on heterogeneity.

**Table 1:** Baseline characteristics

First author (Trial name)	Year	Single or multicentre design (SC/MC)	Tumour stage exclusion criteria	Number of participants LAP/OPEN (*n*)	Female/Male (*n*)	Mean age LAP/OPEN (years)	Mean distance of the tumour from anal verge LAP/OPEN (cm)	Types of surgery	Neoadjuvant treatment LAP/OPEN *n* (%)	Ileostomy LAP/OPEN *n* (%)	Conversion rate *n* (%)
Guillou\Green [18, 19] (CLASICC)	2005	MC	Acute intestinal obstruction	253/128	ND	ND	ND	TME, APR	ND	ND	86 (34)
Braga [20]	2006	SC	T4	83/85	49/119	62.8/65.3	9.1/8.6	TME, APR	14 (16.9)/12 (14.1)	22 (26.5)/21 (24.7)	6 (7.2)
Ng [22]	2008	SC	T4, size >6 cm	51/48	38/61	63.7/63.5	ND	TME	0/0	ND	5 (9.8)
Ng [23]	2009	SC	T4, size >6 cm	76/77	68/85	66.5/65.7	ND	APR	ND	ND	23 (30.3)
Lujan [17]	2009	SC	T4	101/103	78/126	67.8/66.0	5.5/6.2	TME, APR	74 (73.0)/79 (77.0)	48 (47.5)/48 (46.6)	8 (7.9)
Kang\Jeong [16, 24] (COREAN)	2010	MC	T4, M1	170/170	120/220	57.8/59.1	5.6/5.3	TME, APR	170 (100)/170 (100)	138 (81.2)129 (/75.9)	2 (1.2)
van der Pas\Bonjer [9, 15] (COLOR II)	2013	MC	T4	699/345	385/669	66.8/65.8	ND	PME, TME, APR	636 (91.0)/317 (92.0)	243 (34.8)/131 (38.0)	119 (17)
Gong [14]	2012	SC	M1	67/71	60/78	58.4/59.6	ND	TME, APR	ND	ND	2 (3.0)
Ng [21]	2014	SC	T4	40/40	34/46	60.2/62.1	6.9/7.1	TME	ND	20 (50.0)/26 (65.0)	3 (7.5)
Fleshman [10] (ACOSOG Z6051)	2015	MC	T4, M1	240/222	148/314	57.7/57.2	6.1/6.3	TME, APR	236 (98.3)/215 (96.7)	171 (71.3)/165 (74.3)	27 (11.3)
Stevenson [11] (ALaCaRT)	2015	MC	T4	238/237	162/311	65.0/65.0	ND	TME, APR	119 (50.0)/117 (49.4)	68.1/59.5	21 (8.8)

*MC* multicentre, *SC* single centre, *TME* total mesorectal excision (anterior resection), *APR* abdominoperineal resection, *PME* partial (upper) mesorectal excision, *ND* no data, *LAP* laparoscopic approach, *OPEN* open approach

**Fig. 3:**
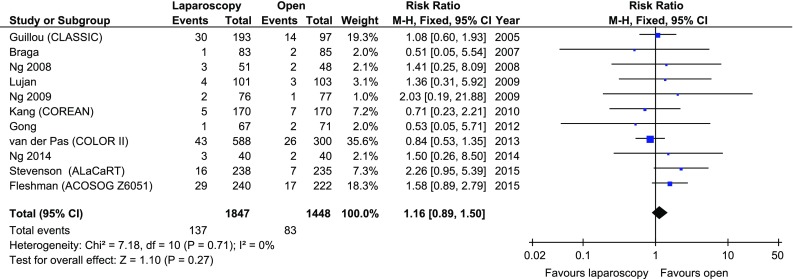
Pooled estimates of involved circumferential resection margins comparing laparoscopy and open surgery. *CI* confidence interval, *df* degrees of freedom, *RR* risk ratio

Involved CRMs were reported in all 11 studies. None of the analysed studies showed differences in CRM status between the laparoscopic and open approach. Overall, there were no statistically significant differences in the number of positive CRMs between the laparoscopic group (137/1847 (7.42%)) and the open group (83/1448 (5.73%)), RR 1.16, 95% CI 0.89–1.50, *p* for effect = 0.27, *p* for heterogeneity = 0.71, *I* ^2^ = 0% (Fig. 3).

**Fig. 4:**
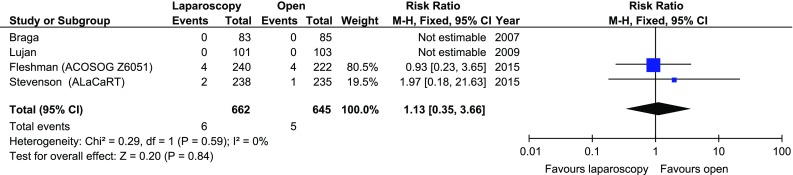
Pooled estimates of involved distal margins comparing laparoscopy and open surgery. *CI* confidence interval, *df* degrees of freedom, *RR* risk ratio

Data on involved distal margins were provided in 4/11 studies. None of the analysed studies showed differences in positive distal margins between the laparoscopic and open approach. The analysis revealed no significant differences in distal margin positivity: 6/662 (0.91%) in the laparoscopic group versus 5/645 (0.78%) in the open group, RR 1.13 95% CI 0.35–3.66, *p* for effect = 0.84, *p* for heterogeneity = 0.59, *I* ^2^ = 0% (Fig. 4).

**Fig. 5:**
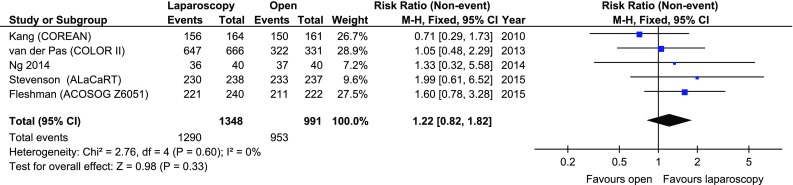
Pooled estimates of completeness of mesorectal excision comparing laparoscopy and open surgery. *CI* confidence interval, *df* degrees of freedom, *RR* risk ratio

The data on the completeness of mesorectal excision were reported in 5/11 papers, involving 2339 patients. In 4 papers, the classification proposed by Nagtegaal et al. was used. In the fifth paper, by Ng et al. [21], mesorectal excision was described as complete or incomplete. In the 4 papers which used complete/nearly complete/incomplete classification, complete mesorectal excision occurred in 1093/1308 (83.56%) of laparoscopic cases and 827/951 (86.96%) of open procedures. Nearly complete excision was recorded in 161/1308 (12.30%) laparoscopic and 89/951 (9.36%) open procedures. Incomplete excision was recorded in 54/1308 (4.13%) laparoscopic and 333/951 (3.47%) open procedures. The meta-analysis of all 5 studies reporting completeness of mesorectal excision (complete was combined with nearly complete and compared with incomplete as in Nagtegaal’s classification) revealed no significant differences among the studies: 1290/1348 (95.69%) versus 953/991 (96.17%), RR 1.22, 95% CI 0.82–1.82, *p* for effect = 0.33, *p* for heterogeneity = 0.6, *I* ^2^ = 0% (Fig. 5).

**Fig. 6:**
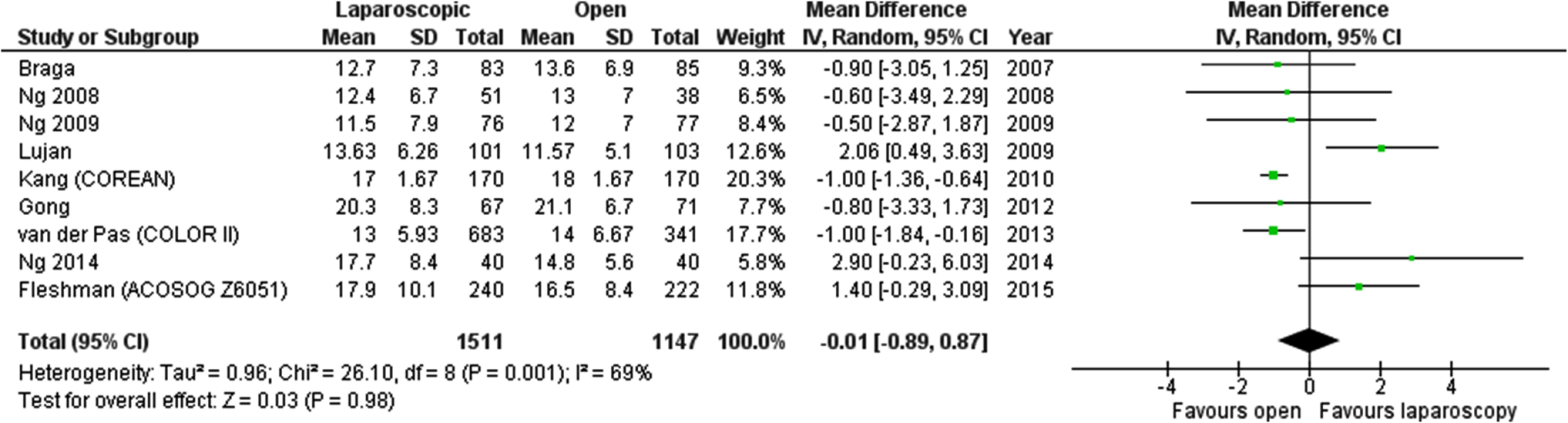
Pooled estimates of harvested lymph node yield comparing laparoscopy and open surgery. *CI* confidence interval, *df* degrees of freedom, *RR* risk ratio

The number of harvested lymph nodes was reported in 9 studies. Kang et al. and van der Pas et al. reported open procedures harvesting a greater number of lymph nodes, whereas Lujan et al. reported the opposite [15, 17, 24]. The remaining studies did not present statistically significant data. Overall, the analysis revealed no statistically significant differences among the studied groups, mean difference = −0.01, 95% CI −0.89 to 0.87, *p* for effect = 0.98, *p* for heterogeneity = 0.001, *I* ^2^ = 69% (Fig. 6).

**Fig. 7:**
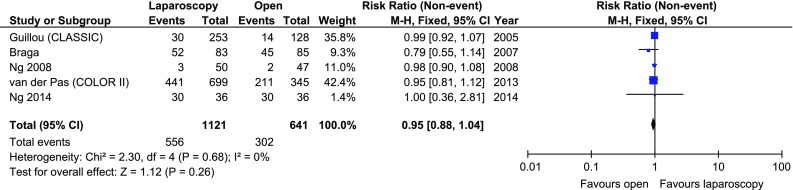
Pooled estimates of 3-year disease-free survival rate comparing laparoscopy and open surgery. *CI* confidence interval, *df* degrees of freedom, *RR* risk ratio

**Fig. 8:**
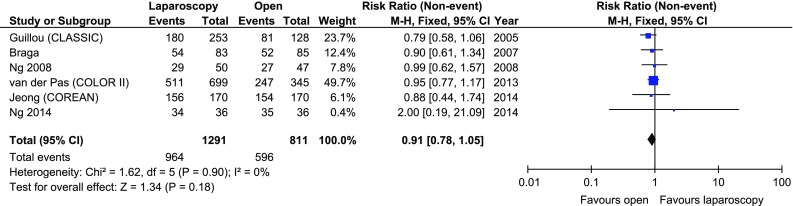
Pooled estimates of 3-year overall survival rate comparing laparoscopy and open surgery. *CI* confidence interval, *df* degrees of freedom, *RR* risk ratio

**Fig. 9:**
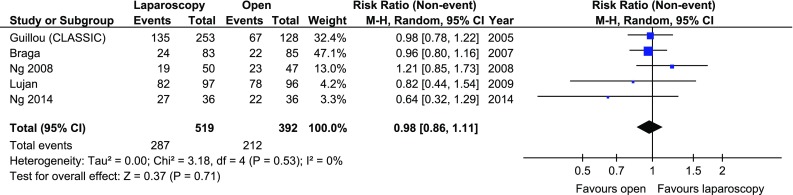
Pooled estimates of 5-year disease-free survival rate comparing laparoscopy and open surgery. *CI* confidence interval, *df* degrees of freedom, *RR* risk ratio

**Fig. 10:**
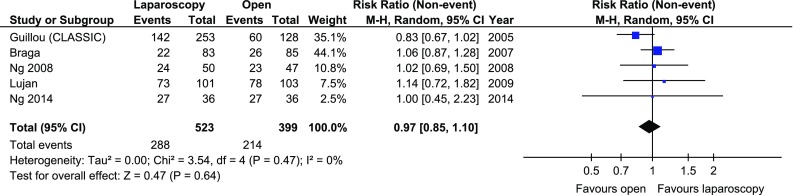
Pooled estimates of 5-year overall survival rate comparing laparoscopy and open surgery. *CI* confidence interval, *df* degrees of freedom, *RR* risk ratio

The disease-free 3-year survival rate was reported in 5 papers, whereas an overall 3-year survival rate was reported in 6. There were no significant variations among the groups [*p* = 0.26 and *p* = 0.18 (Figs. 7, 8)]. Five-year survival and 5-year disease-free survival rates were each reported in by 5 authors. There were no statistically significant differences in 5-year survival rate, *p* = 0.64. No differences were found in terms of disease-free survival either, *p* = 0.71 (Figs. 9, 10).

**Fig. 11:**
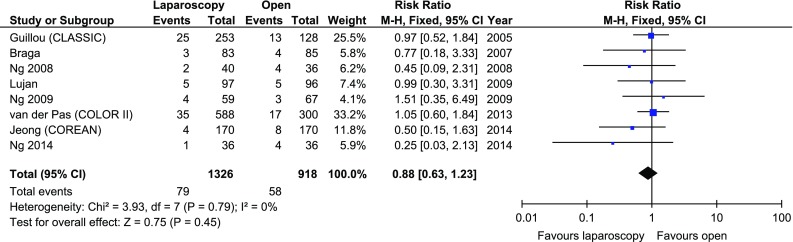
Pooled estimates of local recurrence rate comparing laparoscopy and open surgery. *CI* confidence interval, *df* degrees of freedom, *RR* risk ratio

The local recurrence rate was reported in 8/11 studies. It ranged from 2.35 to 9.88% in the laparoscopic group, and 4.47–11.11% in the open group. There were no statistically significant variations among the studied groups, RR 0.88, 95% CI 0.63–1.23, *p* for effect = 0.45, *p* for heterogeneity = 0.79, *I* ^2^ = 0% (Fig. 11).

## Discussion

We found no difference in circumferential resection margin involvement between laparoscopic and open surgery for rectal cancer. Not difference was found in any other oncological parameter, nor any difference in disease-free or overall survival by 5 years.

The quality of included studies was mostly high and very high. For obvious reasons, none of them blinded the participants and only 5 studies blinded outcome assessors. Although this may create potential bias, one should remember that in surgical RCTs, blinding is either impossible or at the very least, difficult. All analysed studies included groups of patients undergoing laparoscopic resection, no robotic surgery was involved, although there are currently several ongoing trials comparing laparoscopic with robotic surgery registered at clinicaltrials.gov (NCT01736072 (ROLARR), NCT01130233, NCT01985698 (RLOAPR), NCT01591798, NCT02673177 (TRVL), NCT02817126).

The involved CRM rate varied among studies between 1.2 and 15.5% in the laparoscopic group and 1.3–14.43% in the open group. However, there was no overall difference between laparoscopic and open surgery and heterogeneity was low. Differences in CRM involvement between studies may suggest that the quality of surgery varied or (less probably) there were differences in pathologic assessment (there were no pre-operative differences in T stage or use of neoadjuvant therapy between groups). In our meta-analysis, the completeness of total mesorectal excision was similar regardless of the technique used. In a recent meta-analysis by Martínez-Pérez et al. [25], a difference in completeness of mesorectal excision was found favouring open surgery. More studies were included for data extraction in our review and we grouped together ‘complete’ and ‘nearly complete’ resections while Martínez-Pérez et al. compared ‘complete’ resections with a group of flawed excisions (‘nearly complete’ combined with ‘incomplete’). More data are needed to fully establish whether there are differences in overall survival between complete and nearly complete mesorectal excisions.

Abbas et al. [26] highlighted issues that might or might not be relevant to short- and long-term oncologic outcomes in laparoscopic rectal surgery. It was suggested that laparoscopy may be inferior to the open approach due to technical limitations leading to a so-called fulcrum/coning effect during dissection, resulting in positive CRM or incomplete mesorectal excision more often in lower rectal cancers. However, our review we did not find differences in CRM involvement, but only 1 study fully analysed the outcomes in low rectal cancers [15], where statistically different rates of CRM involvement were found in patients with cancer of the lower third of the rectum and interestingly, worse outcomes were observed in the open surgery group (22% involved CRMs in the open group and 9% in laparoscopic group). Certainly, a positive CRM strongly correlates with the height of the tumour [15, 27, 28]. Because of high CRM involvement in low rectal cancers, a novel bottom-up transanal total mesorectal excision has been proposed and currently a multicentre RCT COLOR III trial (NCT02736942) has started to fully assess the oncologic benefits of this approach (estimated primary completion date: May 2020) [29]. There were also differences in conversion rates among studies (1–34%), which confirms the difficulty of the laparoscopic technique and underlines issues with its standardization. High conversion rates are associated with the learning curve and the surgical unit’s experience as well as with tumour stage, which may contribute to worse perioperative outcomes and may also influence survival, although evidence is lacking to draw firm conclusions [30–32].

The number of harvested lymph nodes was similar in the laparoscopic and open group. However, lymph node yield is dependent on many factors such as the tumour itself, the patient, neoadjuvant radiochemotherapy, pathologic assessment [33] and, last but not least, the surgeon [34].

Most importantly, operative technique has no impact on long-term outcomes suggesting that, given the amount of data available further RCTs comparing the laparoscopic and open approach in terms of oncologic outcomes may not be required.

The quality of data in this review has several limitations. In practically all included studies long-term outcomes were not set as a primary endpoint; therefore, most studies were probably underpowered for this parameter. In addition, in most of them involved CRM was used as a universal marker of non-radical operation. However, there is agreement that any involved margin is associated with poor survival. Since distal margins (length and involvement) were not reported in most studies, we were not able to fully assess the R0 resection rate in the analysed groups. According to Parmar et al. [35] in studies involving time to event (survival-type) data, the most appropriate statistics to use are the log hazard ratio and its variance. However, this was not explicitly presented for included studies and we had to compare data after 3 and 5 years post-surgery. Surgeon experience and hospital volume in rectal surgery are important factors influencing outcomes but in this review surgeon experience was not analysed [36–38].

In conclusion, this systematic review with a meta-analysis showed that laparoscopic surgery for rectal cancer is associated with similar short-term and long-term oncologic outcomes compared to open surgery. The oncologic quality of specimens seems comparable regardless of the approach used.

## Electronic supplementary material

Below is the link to the electronic supplementary material.

Supplementary material 1 (TIFF 405 kb)
