# Supplementary figures and images for: There is no difference in outcome between laparoscopic and open surgery for rectal cancer: a systematic review and meta-analysis on short- and long-term oncologic outcomes
Source: Tech Coloproctol. 2017 Aug 9;21(8):595–604. doi: 10.1007/s10151-017-1662-4 (PMC5602007; doi:10.1007/s10151-017-1662-4)

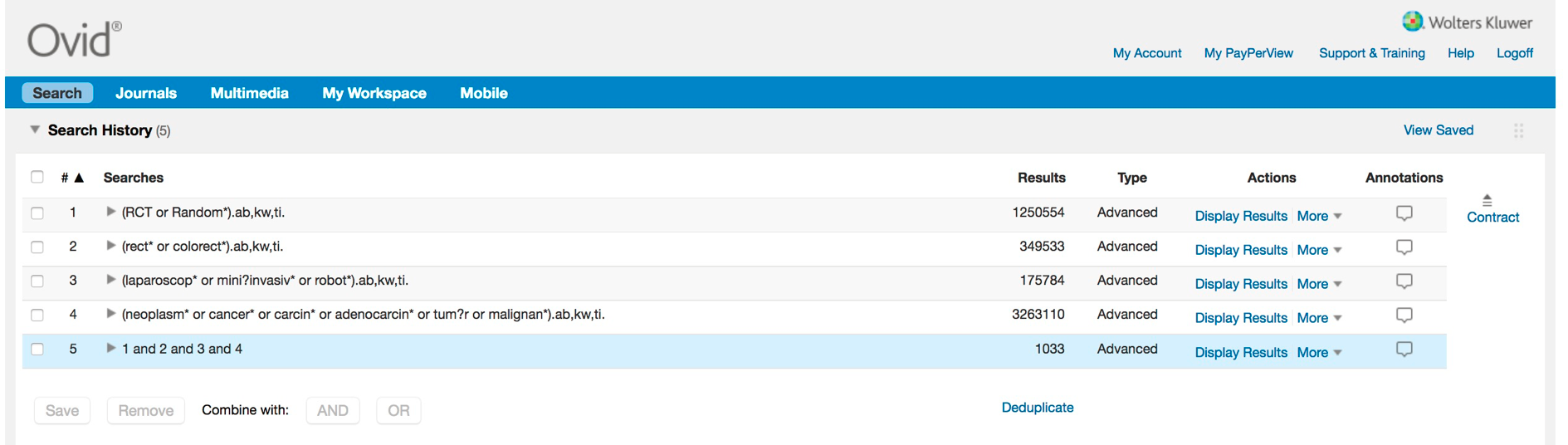

Supplement: Supplementary file 1 — Supplementary material 1 (TIFF 405 kb) [file 10151_2017_1662_MOESM1_ESM.tif]
